# Role of Rutin on Nitric Oxide Synthesis in Human Umbilical Vein Endothelial Cells

**DOI:** 10.1155/2014/169370

**Published:** 2014-06-24

**Authors:** Azizah Ugusman, Zaiton Zakaria, Kien Hui Chua, Nor Anita Megat Mohd Nordin, Zaleha Abdullah Mahdy

**Affiliations:** ^1^Department of Physiology, Faculty of Medicine, Universiti Kebangsaan Malaysia Medical Centre, Jalan Raja Muda Abdul Aziz, 50300 Kuala Lumpur, Malaysia; ^2^Department of Obstetrics and Gynaecology, Universiti Kebangsaan Malaysia Medical Centre, Jalan Yaacob Latif, 56000 Cheras, Kuala Lumpur, Malaysia

## Abstract

Nitric oxide (NO), produced by endothelial nitric oxide synthase (eNOS), is a major antiatherogenic factor in the blood vessel. Oxidative stress plays an important role in the pathogenesis of various cardiovascular diseases, including atherosclerosis. Decreased availability of endothelial NO promotes the progression of endothelial dysfunction and atherosclerosis. Rutin is a flavonoid with multiple cardiovascular protective effects. This study aimed to investigate the effects of rutin on eNOS and NO production in cultured human umbilical vein endothelial cells (HUVEC). HUVEC were divided into four groups: control; oxidative stress induction with 180 *μ*M H_2_O_2_; treatment with 300 *μ*M rutin; and concomitant induction with rutin and H_2_O_2_ for 24 hours. HUVEC treated with rutin produced higher amount of NO compared to control (*P* < 0.01). In the oxidative stress-induced HUVEC, rutin successfully induced cells' NO production (*P* < 0.01). Rutin promoted NO production in HUVEC by inducing eNOS gene expression (*P* < 0.05), eNOS protein synthesis (*P* < 0.01), and eNOS activity (*P* < 0.05). Treatment with rutin also led to increased gene and protein expression of basic fibroblast growth factor (bFGF) in HUVEC. Therefore, upregulation of eNOS expression by rutin may be mediated by bFGF. The results showed that rutin may improve endothelial function by augmenting NO production in human endothelial cells.

## 1. Introduction

Endothelial nitric oxide (NO) possesses various antiatherosclerotic properties. It is involved in the control of vascular tone and blood pressure by causing vasodilatation. NO also inhibits various steps involved in atherogenesis such as oxidation of low density lipoprotein (LDL), platelet aggregation, leucocytes adhesion, and abnormal proliferation of vascular smooth muscle cells [1]. Loss of normal NO production from the endothelium is a cardinal feature of endothelial dysfunction. Based on the vasculoprotective effects of NO, increased endothelial NO synthesis has the potential to be used as a target in the prevention and treatment of cardiovascular diseases [2].

Endothelial nitric oxide synthase (eNOS) is the major enzyme responsible for NO production in the blood vessels [3]. NO synthesis increases when the level and activity of eNOS in the endothelial cells increase [4]. NO synthesis can also be modulated through regulation of eNOS gene expression [5]. Growth factors such as transforming growth factor beta-1 (TGF-*β*1), vascular endothelial growth factor (VEGF), and basic fibroblast growth factor (bFGF) were reported to upregulate eNOS gene expression [6].

Oxidative stress results from the imbalance between the prooxidative and the antioxidative defense mechanisms of the body. The major source of endogenous reactive oxygen species (ROS) is generated from H_2_O_2_ [7], which has been extensively used to induce oxidative stress in* in vitro* experiments [8, 9]. Oxidative stress plays an important role in the pathogenesis of atherosclerosis and cardiovascular diseases by promoting endothelial dysfunction, inflammation, and lipid/lipoprotein peroxidation and lowering NO bioavailability [10]. Loss of normal NO production from the endothelium is a cardinal feature of endothelial dysfunction [11].

Flavonoids are a group of phenolic compounds which can be found naturally in plants. Epidemiological studies indicate that increased intake of dietary flavonoids is associated with a decrease in the risk of cardiovascular diseases [12]. The cardiovascular protective effects of flavonoids may be mediated by multiple mechanisms. One possible pathway is by increasing eNOS expression and NO synthesis. Increased NO, produced by higher levels of eNOS, might in turn inhibit pathways leading to endothelial dysfunction and atherosclerosis [13].

Rutin (3,3′,4′,5,7-pentahydroxyflavone-3-rhamnoglucoside) is a flavonoid which can be found in buckwheat, apple, green tea,* Betula pendula* leaves, and other sources [14, 15]. It has antioxidant [16], anti-inflammatory [17], and antiplatelet [18] activities. Rutin supplementation causes lowering of blood pressure in rats with metabolic syndrome [19] and relaxation of rats' aortic rings [20].

Rutin is one of the active compounds found in* Piper sarmentosum* leaves [15].* Piper sarmentosum* is a creeping terrestrial herbaceous plant that belongs to the* Piperaceae* family. It is commonly found in the tropical and subtropical regions of the world, such as the Asian and South East Asia regions [21].* Piper sarmentosum* had been shown to promote NO production in HUVEC [22]. However, the active compound responsible for the effect remains unclear. Therefore, the present study was designed to look into the effects of rutin on the eNOS system and NO synthesis in HUVEC. The results of the present study may help in the prevention and treatment of endothelial dysfunction which is linked to various cardiovascular diseases. Furthermore, beneficial results from this study will also add to the scientific basis of using* Piper sarmentosum* as a supplement for cardiovascular health.

## 2. Materials and Methods

### 2.1. Materials

Rutin (purity 95%), hydrogen peroxide (H_2_O_2_), and ethidium bromide were purchased from Sigma (St. Louis, USA). Collagenase type I was purchased from Gibco-Invitrogen Corp. (Grand Island, USA). Medium 200 and low serum growth supplement (LSGS) were purchased from Cascade Biologics (Grand Island, USA). TRI Reagent and polyacryl carrier were purchased from Molecular Research Center (Cincinnati, USA). RNase and DNase free water and SuperScript III First-Strand Synthesis SuperMix were purchased from Invitrogen (Carlsbad, USA). IQ SYBR Green Supermix was purchased from Bio-Rad (Hercules, USA). Quantikine human eNOS ELISA kit was purchased from R&D Systems Inc. (Minneapolis, USA). Calbiochem nitric oxide synthase assay kit was purchased from EMD Chemicals (Darmstadt, Germany). Bioxytech nitric oxide assay kit was purchased from OxisResearch (Portland, USA). Procarta cytokine kit was purchased from Panomics (Fremont, USA).

### 2.2. Cell Culture and Treatment Protocol

Human umbilical cords were obtained under sterile condition from labour room in Hospital Kuala Lumpur. Written consent was obtained from each subject and the present study was approved by the Ethical Research Committee of Universiti Kebangsaan Malaysia Medical Center (approval code: FF-092-2010). HUVEC were obtained from umbilical cord veins by 0.1% collagenase type I digestion. Cells were grown in medium 200 supplemented with LSGS at 37°C in a humidified atmosphere of 5% CO_2_ and 95% air. HUVEC were confirmed by the typical endothelial cell cobblestone morphology and the positive expressions of von Willebrand factor and CD31 in immunocytochemistry. The culture medium was changed every other day until the cells reached confluence. HUVEC from passage 3 at 80% confluency were used for experiments. The cells were divided into four groups as follows: control; oxidative stress induction with 180 *μ*M H_2_O_2_; treatment with 300 *μ*M rutin only; and concomitant induction with 300 *μ*M rutin and 180 *μ*M H_2_O_2_. All treatments were given for 24 hours. The dose of H_2_O_2_ used was based on the IC_50_ of H_2_O_2_ adopted from a previous study [22] while 300 *μ*M rutin was used as it significantly increased HUVEC viability by almost 50 percent when induced with 180 *μ*M H_2_O_2_ [15].

### 2.3. Quantitative Reverse Transcription Polymerase Chain Reaction (qPCR) for Analysis of eNOS, TGF*β*1, bFGF, and VEGF mRNA Expression

Following treatment for 24 hours, total ribonucleic acid (RNA) from HUVEC was extracted using TRI Reagent as previous research protocol [23]. Polyacryl carrier was added to precipitate the total RNA. Extracted RNA pellet was then washed with 75% ethanol and dried prior to dissolving it in RNase and DNase free water. Extracted total RNA was assessed for its purity and quantity using Nanodrop ND-100 spectrophotometer (Wilmington DE, USA) and stored at −80°C before use. Complimentary DNA (cDNA) was synthesized using SuperScript III First-Strand Synthesis SuperMix. A total of 20 *μ*L of volume reaction which consisted of 10 *μ*L of 2X RT reaction mix, 2 *μ*L of RT enzyme, 5 *μ*L of total RNA, and 3 *μ*L of DEPC-treated water was incubated at 25°C for 10 minutes for primer annealing then at 50°C for 30 minutes for reverse transcription. Following this, the reaction was terminated at 85°C for 5 minutes, chilled on ice for 1 minute, and 1 *μ*L of* E. coli* RNase H was added to the mixture. The cDNA was further incubated at 37°C for 20 minutes and stored at −20°C until use. Subsequently, qPCR was carried out to determine the mRNA expression level of eNOS, TGF*β*1, bFGF, and VEGF. Glycerylaldehyde-3-phosphate dehydrogenase (GAPDH) was used as the reference gene. Primer 3 software was used to design the primers from NIH GenBank database. The primer sequences for eNOS, TGF*β*1, bFGF, and VEGF were listed in Table 1. The qPCR reaction was performed with 1 *μ*L of cDNA, 5 *μ*M of each forward and reverse primer and 12.5 *μ*L of IQ SYBR Green Supermix in BioRad iCycler (Bio-Rad, USA) with reaction profile of: 40 cycles of 95°C (10 seconds) and 61°C (30 seconds). The reaction kinetic of each primer set and protocol was verified with melting profile and product size was further confirmed with 2% agarose gel electrophoresis stained with ethidium bromide. The threshold cycle (CT) value was determined and the relative mRNA expression of eNOS, TGF*β*1, bFGF, and VEGF was calculated as follows: 2^ΔΔCT^ with ΔΔCT = CT  GAPDH − CT gene of interest.

### 2.4. Enzyme-Linked Immunosorbent Assay (ELISA) for eNOS Protein Analyses

eNOS protein level of the cultured HUVEC was determined by using Quantikine human eNOS ELISA kit. HUVEC were washed with phosphate-buffered saline (PBS) twice, manually scraped from the culture flask, and lysed with 400 *μ*L of lysis buffer. The assay was performed using 100 *μ*L of the cell lysate. The cell lysate was pipetted into the 96-well plate so that any eNOS present would be bound to the immobilized antibody in the plate. After washing away any unbound substances, eNOS conjugate was added to the wells. This was followed by addition of substrate solution and stop solution. The optical density of each well was determined at 450 nm using an ELISA microplate reader.

### 2.5. Determination of eNOS Activity

eNOS activity was determined by using Calbiochem nitric oxide synthase assay kit. The principle of this assay was based on the measurement of nitrite produced by eNOS in the sample in a timed reaction. HUVEC were scraped from the culture flask, homogenized in PBS, and centrifuged at 10,000 g for 20 minutes. Then, the cell lysate in the supernatant solution was filtered through a 0.45 *μ*m filter prior to ultracentrifugation at 100,000 g for 15 minutes. A total of 40 *μ*L of the cell lysate was diluted with 20 *μ*L of assay buffer. Then the samples were mixed with NADPH, nitrate reductase, cofactor preparation solution, and lactate dehydrogenase (LDH). Total nitrite was measured at 540 nm absorbance by reaction with Griess reagents (sulfanilamide and naphthalene-ethylenediamine dihydrochloride). Concentration of nitrite in the sample was calculated using a standard curve. The eNOS activity was expressed as nmol of nitrite/min per mL of sample.

### 2.6. Determination of Endothelial Nitric Oxide Production

Production of NO by HUVEC was measured as its stable oxidation product; nitrite, using Bioxytech nitric oxide assay kit. Briefly, 50 *μ*L of the culture medium was diluted with 35 *μ*L assay buffer and mixed with 10 *μ*L nitrate reductase and 10 *μ*L NADH. Following 20 minutes of incubation to convert nitrate to nitrite, total nitrite was measured at 540 nm absorbance by reaction with Griess reagents (sulfanilamide and naphthalene-ethylenediamine dihydrochloride).

### 2.7. Luminex Assay for TGF*β*1, bFGF, and VEGF Protein Analyses

TGF*β*1, bFGF, and VEGF protein levels of the cultured HUVEC were obtained using Procarta cytokine kit in 96-well plate ELISA-based formats according to manufacturer's instructions. The sensitivity of the assay (limit of detection) was 1 pg/mL/cytokine [24]. Following incubation with antibody-conjugated beads, detection antibodies, and streptavidin-phycoerythrin (SA-PE) complexes, samples were analyzed with Luminex 100 instrument (Luminex Corporation). Fluorescence signals were collected and data was expressed in pg/mL using internal standards as the mean of three individual experiments done in triplicate.

### 2.8. Statistical Analysis

Data was tested for normality using Kolmogorov-Smirnov test and all variables were normally distributed. Data was expressed as mean ± SEM. Statistical analysis between two groups was performed using paired Student's *t*-test using SPSS version 17.0 software. Values of *P* < 0.05 were considered statistically significant.

## 3. Results

### 3.1. Effect of Rutin on eNOS mRNA Expression in HUVEC

**Figure 1:**
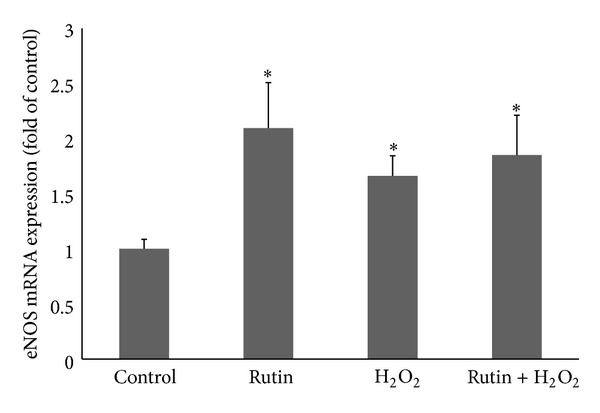
Bar chart showing eNOS mRNA expression in control, rutin, H_2_O_2_, and rutin + H_2_O_2_ groups. Values are expressed as means ± SEM of *n* = 8. *P < 0.05 versus control.

eNOS mRNA expression in HUVEC treated with rutin increased by 2.1-fold compared to the control group (*P* < 0.05) (Figure 1). In the oxidative stress-induced group, HUVEC treated with H_2_O_2_ showed a significant increase in eNOS mRNA expression by 1.6 times compared to the control group (*P* < 0.05). Concomitant treatment of HUVEC with both rutin and H_2_O_2_ caused an increase in eNOS mRNA expression by 1.8 times compared to the control group (*P* < 0.05).

### 3.2. Effect of Rutin on eNOS Protein Level in HUVEC

**Figure 2:**
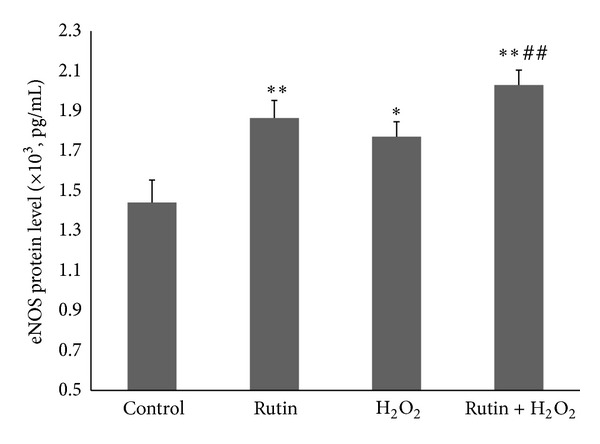
Bar chart showing eNOS protein level in control, rutin, H_2_O_2_, and rutin + H_2_O_2_ groups. Values are expressed as means ± SEM of *n* = 8. *P < 0.05 versus control; **P < 0.01 versus control; and ^##^ *P* < 0.01 versus H_2_O_2_.

eNOS protein level in HUVEC treated with rutin (1.864 ± 0.088 × 10^3^ pg/mL) increased significantly (*P* < 0.01) compared to the control (1.441 ± 0.113 × 10^3^ pg/mL) (Figure 2). The H_2_O_2_-induced group (1.771 ± 0.075 × 10^3^ pg/mL) also showed a significant increase in eNOS protein level compared to the control (*P* < 0.05). HUVEC induced with both rutin and H_2_O_2_ (2.029 ± 0.075 × 10^3^ pg/mL) showed a significant increase in eNOS protein level compared to the control group (*P* < 0.01) and H_2_O_2_ group (*P* < 0.01).

### 3.3. Effect of Rutin on eNOS Activity in HUVEC

**Figure 3:**
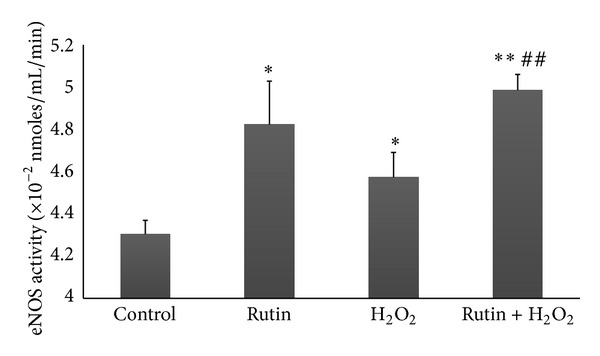
Bar chart showing eNOS activity in control, rutin, H_2_O_2_, and rutin + H_2_O_2_ groups. Values are expressed as means ± SEM of *n* = 8. *P < 0.05 versus control; **P < 0.01 versus control; and ^##^ *P* < 0.01 versus H_2_O_2_.

eNOS activity in HUVEC treated with rutin (4.823 ± 0.205 × 10^−2^ nmoles/mL/min) increased significantly (*P* < 0.05) compared to the control (4.304 ± 0.065 × 10^−2^ nmoles/mL/min) (Figure 3). The H_2_O_2_-induced group (4.573 ± 0.118 × 10^−2^ nmoles/mL/min) also showed a significant increase in eNOS activity compared to the control (*P* < 0.05). HUVEC induced with both rutin and H_2_O_2_ (4.986 ± 0.074 × 10^−2^ nmoles/mL/min) showed a significant increase in eNOS activity compared to the control group (*P* < 0.01) and the H_2_O_2_ group (*P* < 0.01).

### 3.4. Effect of Rutin on NO Production in HUVEC

**Figure 4:**
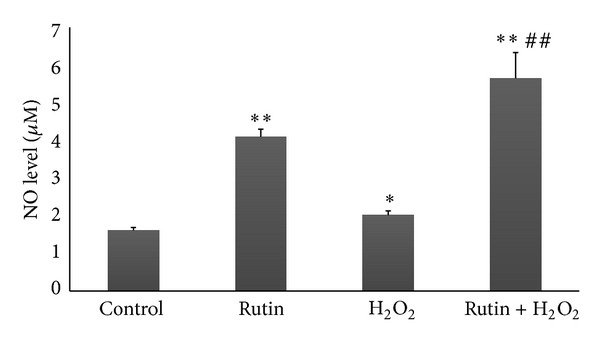
Bar chart showing NO level in control, rutin, H_2_O_2_, and rutin + H_2_O_2_ groups. Values are expressed as means ± SEM of *n* = 8. *P < 0.05 versus control; **P < 0.01 versus control; and ^##^ *P* < 0.01 versus H_2_O_2_.

There was a significant increase (*P* < 0.01) in the level of NO produced by HUVEC treated with rutin (4.095 ± 0.203 *μ*M) compared to the control (1.605 ± 0.08 *μ*M) (Figure 4). HUVEC induced with H_2_O_2_ produced higher amount of NO (2.01 ± 0.115 *μ*M) compared to the control (*P* < 0.01). The highest level of NO was produced by HUVEC treated with both rutin and H_2_O_2_ (5.65 ± 0.683 *μ*M) whereby this increase was significant compared to the control group (*P* < 0.01) and the H_2_O_2_ group (*P* < 0.01).

### 3.5. Effects of Rutin on TGF*β*1, bFGF, and VEGF mRNA Expression in HUVEC

**Figure 5:**
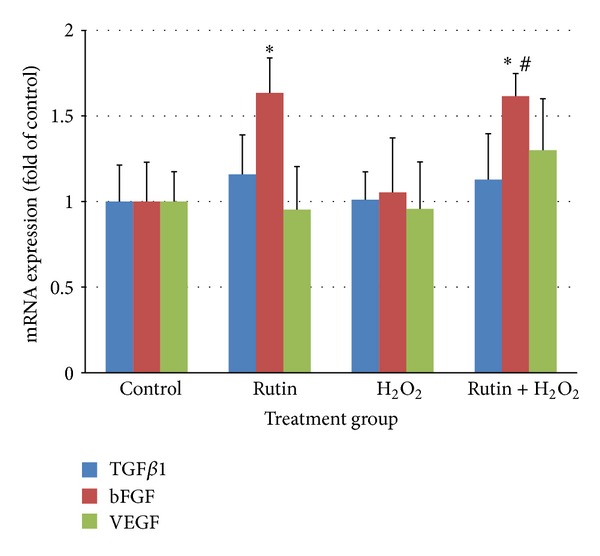
Bar chart showing TGF*β*1, bFGF, and VEGF mRNA expression in control, rutin, H_2_O_2_, and rutin + H_2_O_2_ groups. Values are expressed as means ± SEM of *n* = 8. *P < 0.05 versus control; ^#^ *P* < 0.05 versus H_2_O_2_.

bFGF mRNA expression in HUVEC treated with rutin increased significantly (*P* < 0.05) by 1.6 times compared to the control (Figure 5). HUVEC treated with both rutin and H_2_O_2_ also showed higher level of bFGF mRNA expression compared to the control (*P* < 0.01) and the H_2_O_2_ (*P* < 0.01) groups. There was no significant difference in the mRNA expression of TGF*β*1 and VEGF.

### 3.6. Effects of Rutin on TGF*β*1, bFGF, and VEGF Protein Level in HUVEC

**Figure 6:**
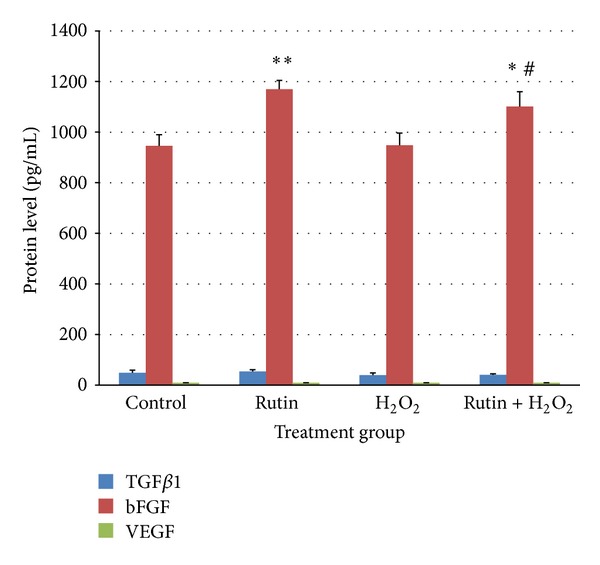
Bar chart showing TGF*β*1, bFGF, and VEGF protein level in control, rutin, H_2_O_2_, and rutin + H_2_O_2_ groups. Values are expressed as means ± SEM of *n* = 8. *P < 0.05 versus control; **P < 0.01 versus control; and ^#^ *P* < 0.05 versus H_2_O_2_.

bFGF protein level in HUVEC treated with rutin (1169.715 ± 34.663 pg/mL) increased significantly (*P* < 0.01) compared to the control (946.198 ± 44.043 pg/mL) (Figure 6). HUVEC treated with both rutin and H_2_O_2_ also showed higher level of bFGF protein compared to the control (*P* < 0.05) and H_2_O_2_ (947.696 ± 48.933 pg/mL) (*P* < 0.05) groups. There was no significant difference in the protein level of TGF*β*1 and VEGF. The increase in bFGF protein level was in parallel with the increase in bFGF mRNA expression (Figure 5).

## 4. Discussion

Results showed that rutin increased NO production by HUVEC. Rutin also caused upregulation of eNOS mRNA expression and increase in eNOS protein level and eNOS activity. The increase in eNOS mRNA expression caused more eNOS protein to be synthesized. The higher amount of eNOS protein led to a higher level of eNOS activity. This resulted in an increase in the NO production by HUVEC. eNOS protein level was significantly increased in the combined rutin + H_2_O_2_ group compared to the H_2_O_2_ group (*P* < 0.01) (Figure 2). However, eNOS mRNA expression was not significantly increased when comparing between these two groups (Figure 1). This could be due to the level of eNOS protein in the rutin + H_2_O_2_ group which was high enough to inhibit eNOS mRNA expression via negative feedback mechanism [25].

Even though H_2_O_2_ treatment alone increased NO production, the combined treatment of HUVEC with rutin and H_2_O_2_ significantly increased NO production compared to both control and H_2_O_2_ groups. The results suggested that rutin may improve endothelial function by augmenting NO production in human endothelial cells.* Piper sarmentosum* was reported to enhance endothelial NO synthesis [22]. Since rutin is one of the major flavonoids found in* Piper sarmentosum* [15], it may play a role in modulating the stimulatory effect of* Piper sarmentosum* on NO production.

An earlier study reported rutin to cause vasorelaxation in potassium- and phenylephrine-induced contractions in isolated rat thoracic aorta [20]. The vasorelaxant effect of rutin involved the release of NO from the endothelium as pretreatment with NO synthase inhibitor, and NG-nitro-L-arginine methyl ester (L-NAME) attenuated the response [20]. Rutin-treated rats with metabolic syndrome had lower blood pressure and improved endothelial function. The hypotensive effect of rutin could be mediated by the increase in NO [19].

Oxidative stress can contribute to the development and progression of atherosclerosis by promoting endothelial dysfunction, inflammation, and lipid peroxidation and lowering NO bioavailability [10]. In the present study, oxidative stress induction in HUVEC by addition of 180 *μ*M H_2_O_2_ increased eNOS mRNA expression, eNOS protein level eNOS activity, and NO level (Figures 1, 2, 3, and 4). The responses to H_2_O_2_ in this study were in accordance with earlier reports [10, 26]. NO level was higher in the H_2_O_2_-treated group compared to the control group. This may be due to induction of NO production by H_2_O_2_ as part of the self-protective mechanism of the cells. The dose of H_2_O_2_ used in this study was not lethal to HUVEC, therefore the cells were still able to increase its endogenous NO production when being challenged by H_2_O_2_. However, H_2_O_2_ also caused oxidative destruction of the synthesized NO, which explained why the increase in NO in the H_2_O_2_-treated group was not as high as the other groups like rutin and the combined rutin and H_2_O_2_ groups (Figure 4). H_2_O_2_-upregulated eNOS expression represents a self-protective mechanism of the endothelial cells to maintain NO bioactivity under conditions of enhanced oxidative stress. H_2_O_2_ also increases eNOS activity by inducing changes in the phosphorylation status of the enzyme [27].

Antioxidants are well known to enhance the biological actions of NO by protecting NO against oxidative destruction by ROS [27]. Rutin was shown to exhibit antioxidant properties [16] and cytoprotective effects against H_2_O_2_-induced oxidative cell damage [15]. Thus, rutin may directly protect NO from oxidative destruction by H_2_O_2_. Rutin also enhanced NO production in HUVEC through increase in eNOS mRNA expression and protein synthesis as well as the enzyme activity (Figures 1, 2, and 3). Thus, all these mechanisms contributed to the increase in the NO level.

In a previous study, rutin significantly attenuated H_2_O_2_-induced cytotoxicity and apoptosis in HUVEC in a concentration-dependant manner [28]. Reactive oxygen species (ROS) (superoxide, H_2_O_2_, and hydroxyl radicals) are potent intracellular oxidants which were proposed as critical regulators of apoptosis [29]. Reduced glutathione (GSH) is a major antioxidant that protects cells from oxidative stress by scavenging peroxides in the mitochondria [30]. H_2_O_2_ may cause endothelial cell injury by inducing mitochondrial dysfunction which includes loss of mitochondrial membrane potential [31]. Rutin protected HUVEC against H_2_O_2_-induced cytotoxicity by decreasing the intracellular ROS level, increasing the intracellular GSH, and restoring the mitochondrial membrane potential, along with the capacity of suppressing endothelial cell apoptosis [28].

Incubation of HUVEC with 50, 100, and 200 *μ*M H_2_O_2_ for one hour was able to stimulate inducible nitric oxide synthase (iNOS) mRNA and protein [32]. Therefore, the NO produced by the H_2_O_2_-treated group may be also contributed to iNOS apart from eNOS (Figure 4). Previous study showed that rutin suppressed iNOS gene transcription and NO production in lipopolysaccharide-stimulated RAW 264.7 macrophages [33]. Rutin also inhibited iNOS activity in the kidneys of rats during ischemia-reperfusion injury [34].

Results of the present study also showed that rutin increased bFGF mRNA and protein expression (Figures 5 and 6). There were no significant changes in mRNA and protein expression of TGF*β*1 and VEGF. Previous studies showed that bFGF caused an increase in the eNOS expression* in vitro* and* in vivo* [35]. Since rutin increased the expression of eNOS and bFGF, it is suggested that upregulation of eNOS expression by rutin may be mediated by bFGF. However, in the present study, the data was not enough to conclude the role of TGF*β*1, VEGF, and bFGF in rutin-induced eNOS expression and NO production. We advocate parallel experiments using specific inhibitor or siRNA in future.

Incubation of bovine aortic endothelial cells with bFGF leads to increased eNOS mRNA expression, eNOS protein level, and eNOS activity [36]. Besides, bFGF also stimulated the expression of eNOS mRNA and protein in ovine fetoplacental artery endothelial cells [37]. Intravenously administered bFGF lowered blood pressure by causing systemic vasodilatation [38]. bFGF-induced vasodilatation was attenuated by coadministration of L-NAME; showing that the vasodilatation was mediated by NO-dependent mechanism [39]. Blood vessels of spontaneously hypertensive rats had low bFGF content [40]. Restoration of bFGF to physiological levels either by systemic administration or by* in vivo* gene transfer significantly augmented the number of endothelial cells with positive immunostaining for eNOS, corrected hypertension, and improved vasorelaxation [40].

**Figure 7:**
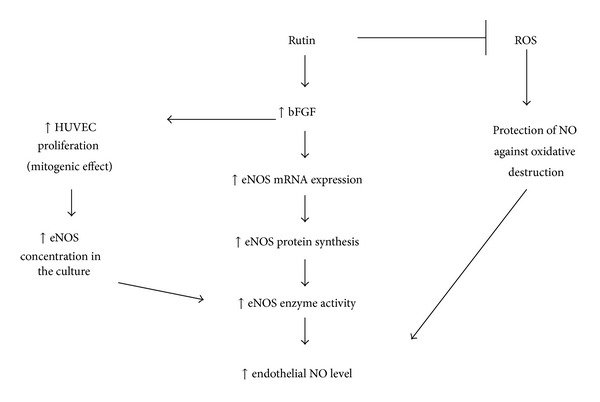
Schematic representation of mechanisms involved in rutin-mediated NO synthesis in HUVEC.

bFGF has a mitogenic effect whereby it may stimulate proliferation of various cells including endothelial cells [41]. Rutin stimulated bFGF expression and bFGF had a mitogenic effect on endothelial cells. This mitogenic effect may lead to the increase in HUVEC culture proliferation. Increase in the number of endothelial cells will cause higher concentration of eNOS in the culture. This may lead to increase in eNOS activity and subsequently more NO production by HUVEC. The mechanisms involved in rutin-promoting effects on endothelial NO production were summarized in Figure 7.

bFGF stimulates eNOS expression via activation of the mitogen-activated protein kinase (MAPK) p44 and p42 pathways or also known as extracellular signal-regulated kinases 1/2 (ERK or ERKs). Active ERK phosphorylates several cytosolic and membrane-bound targets and, upon translocation from the cytoplasm into the nucleus, activates different transcription factors thus also regulating gene transcription [42]. The response to bFGF started when bFGF binds to its receptor which contains tyrosine kinase domain. This may lead to phosphorylation and activation of MAPK p44 and p42 by MAPK kinase in the cytosol. MAPK p44 and p42 will then be translocated from cytosol to nucleus where it stimulates eNOS transcription [37, 43–45]. This activation was inhibited by PD 98059, a specific MAPK kinase inhibitor [37]. Since, rutin increases bFGF which, in turn, increases ERK activity, it may be postulated that rutin may also change ERK kinetic and its intracellular localization between the cytosol and the nucleus.

However, activation of eNOS in Chinese hamster ovary (CHO)-K1 cells is independent of the MAPK cascade [46]. In its inactive form, eNOS is bound to caveolin 1 in caveolae at the plasma membrane. Dissociation of eNOS from caveolin 1 and its translocation to the cytosol are important steps in eNOS activation [47]. In CHO-K1 cells, bFGF activates sphingomyelinase to synthesize ceramide, which, in turn, allows the dissociation of eNOS from caveolin 1 and its translocation to cytosol where it catalyzes the synthesis of NO [46].

The results also showed that there was no significant increase in the VEGF mRNA and protein expression in response to H_2_O_2_ treatment (Figures 5 and 6). However, previous study showed dose-dependent increase in the expression of VEGF in HUVEC treated with 6.25–50 *μ*M H_2_O_2_ [48]. Experimental results obtained with different HUVEC isolates cannot easily be compared to each other because of their different donor origin [49]. Watson et al. [50] reported, for example, that the response to interleukin-8 stimulation is different among several commercially available HUVEC and “home-isolated” primary cultured HUVEC. Different growth media and growth conditions may also contribute to the variations [50].

## 5. Conclusion

The results of the present study showed that rutin promoted NO production in HUVEC by inducing eNOS mRNA expression, protein synthesis, and eNOS activity. Rutin's stimulatory effect on eNOS expression may be mediated by bFGF.

## Figures and Tables

**Table 1:** List of primers for qPCR analysis.

mRNA target	Genbank accession number	Primer sequence	PCR product size (bp)
GAPDH	NM_002046	F: tcc ctg agc tga acg gga ag R: gga gga gtg ggt gtc gct gt	217
eNOS	NM_000603	F: ttt gcc ctt atg gat gtg aag R: cgc atc aaa gaa agc tca gtc	139
TGF*β*1	NM_000358	F: aac aca tca gag ctc cga gaa R: gag gta tcg cca gga att gtt	141
VEGF	NM_001033756	F: ccc act gag gag tcc aac at R: aaa tgc ttt ctc cgc tct ga	173
bFGF	NM_002006	F: ccg tta cct ggc tat gaa gg R: act gcc cag ttc gtt tca gt	158
